# Changing the core of transcription

**DOI:** 10.7554/eLife.03575

**Published:** 2014-07-08

**Authors:** Katherine A Jones

**Affiliations:** 1 **Katherine A Jones** is in the Regulatory Biology Laboratory, The Salk Institute for Biological Studies, La Jolla, United Statesjones@salk.edu

**Keywords:** transcription factors, neuronal gene expression, stem cells, motor neurons, TBP-associated factor TAF, neuronal enhancers, mouse

## Abstract

Different members of the TAF family of proteins work in differentiated cells, such as motor neurons or brown fat cells, to control the expression of genes that are specific to each cell type.

**Related research articles** Herrera FJ, Yamaguchi T, Roelink H, Tjian R. 2014. Core promoter factor TAF9B regulates neuronal gene expression. *eLife* **3**:e02559. doi: 10.7554/eLife.02559; Zhou H, Wan B, Grubisic I, Kaplan T, Tjian R. 2014. TAF7L modulates brown adipose tissue formation. *eLife* **3**:e02811. doi: 10.7554/eLife.02811

**Image** Motor neurons (green) being grown in vitro

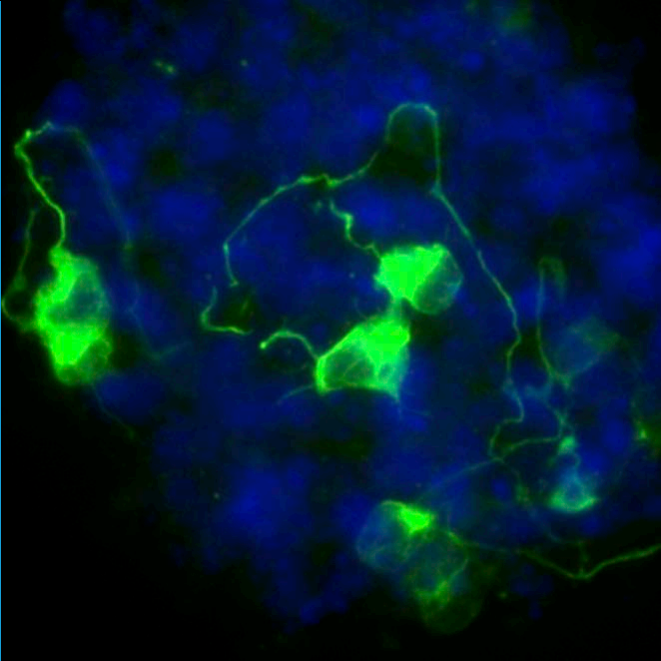

When an organism is developing, different genes are expressed at different times and the pattern of gene expression can often change abruptly. Expressing a gene involves multiple steps: the DNA must be transcribed into a molecule of messenger RNA, which is then translated into a protein. The mechanisms that start the transcription of protein-coding genes in rapidly growing cells are reasonably well understood: two types of proteins—DNA-binding activators and general transcription factors—cooperate to recruit an enzyme called RNA polymerase, which then transcribes the gene (Kadonaga, 2012). These proteins bind to a region of the gene called the promoter, which is upstream from the protein-coding region of the gene.

TATA-binding protein is a general transcription factor that binds to certain sequences of DNA bases found within promoters. TATA-binding protein (or related proteins) also bind to 14 TATA-binding protein associated factors (TAFs). These factors are included into two different protein complexes called TFIID and SAGA (Müller et al., 2010). which, in budding yeast, can recruit TATA-binding protein to gene promoters (Basehoar et al., 2004). There are, however, few hard-and-fast rules in this process: not all genes require all of the general transcription factors, and some genes require both TFIID and SAGA complexes.

Although the steps that are required to switch on genes when cells are rapidly dividing are fairly well known, the same is not true for cells that are differentiating into specialised cell types. In these cells, many transcription factors are down-regulated and the entire pattern of gene expression changes dramatically. Moreover, certain TAFs are strongly up-regulated during differentiation. The core transcriptional machinery is essentially rebuilt at the genes that are expressed in differentiated cells; however, how this occurs is a fascinating and largely unresolved question.

Over the years Robert Tjian of the University of California Berkeley and co-workers have illuminated how individual TAFs can affect how a cell differentiates in different contexts (Figure 1). Now, in *eLife*, Francisco Herrera of UC Berkeley and co-workers—including Teppei Yamaguchi, Henk Roelink and Tjian—have identified a critical role for a TAF called TAF9B in the expression of genes in motor neurons (Herrera et al., 2014). When mouse embryonic stem cells were coaxed, in vitro, into becoming the motor neurons, TAF9B was selectively up-regulated. Herrera et al. also observed high levels of TAF9B in the spinal cord tissue of newborn mice. Deleting the gene for TAF9B in mouse embryonic stem cells revealed that this TAF is not needed for the growth of stem cells, nor is it required for the expression of genes that prevent differentiation: both of these processes are known to be highly-dependent upon the TFIID complex (Pijnappel et al., 2013). However, genes that would normally be expressed specifically in neurons were not up-regulated when cells without the *TAF9B* gene started to specialise.

**Figure 1.**
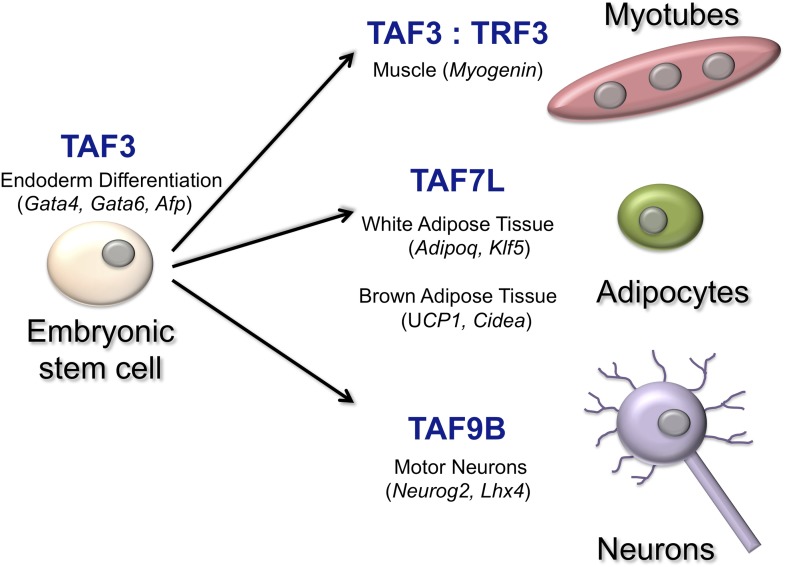
TATA-binding protein associated factors (TAFs) regulate transcription in specific cell types. TAF3, for example, works with another transcription factor to regulate the expression of genes that are critical for the differentiation of the endoderm in the early embryo (Liu et al., 2011). TAF3 also forms a complex with the TATA-related factor, TRF3, to regulate *Myogenin* and other muscle-specific genes to form myotubes (Deato et al., 2008). TAF7L interacts with another transcription factor to activate genes involved in the formation of adipocytes (‘fat cells’) and adipose tissue (Zhou et al., 2013; Zhou et al., 2014). Finally, TAF9B is a key regulator of transcription in motor neurons (Herrera et al., 2014). The names of some of the genes regulated by the TAFs are shown in brackets.

Herrera et al. identified numerous genes that can only be switched on when the TAF9B protein is present, which means that it joins a growing list of TAF proteins that are dedicated to controlling the expression of genes in specialised cell types. Further studies revealed that TAF9B activates neuron-specific genes by binding to sites that reside outside of these genes' core promoters. Further, many of these sites were also bound by a master regulator of motor neuron-specific genes. Herrera et al. found that TAF9B predominantly associates with the SAGA complex, rather than the TFIID complex, in the motor neuron cells. Mice in which the gene for TAF9B had been deleted had less neuronal tissue in the developing spinal cord. Moreover, the genes that are involved in forming the branches of neurons were not properly regulated in these mice.

Recently, in another *eLife* paper, Tjian and co-workers at Berkeley, Fudan University and the Hebrew University of Jerusalem—including Haiying Zhou as first author, Bo Wan, Ivan Grubisic and Tommy Kaplan—reported that another TAF protein, called TAF7L, works as part of the TFIID complex to up-regulate genes that direct cells to become brown adipose tissue (Zhou et al., 2014).

Whilst most of the fat tissue in humans is white adipose tissue, which contains cells that store fatty molecules, some is brown adipose tissue, or ‘brown fat’, that instead generates heat. When TAF7L promotes the differentiation of brown fat, it up-regulates genes that are targeted by a transcription factor called PPAR-γ; last year it was shown that this transcription factor also promotes the differentiation of white adipose tissue (Zhou et al., 2013).

Mice without the *TAF7L* gene had 40% less brown fat than wild-type mice, and also grew too much skeletal muscle tissue. TAF7L was specifically required to activate genes that control how brown fat develops and functions. Thus TAF7L expression appears to shift the fate of a stem cell towards brown adipose tissue, potentially at the expense of skeletal muscle, as both cell types develop from the same group of stem cells. When stem cells with less TAF7L than normal are differentiated in vitro, they yield more muscle than fat cells. Conversely, cells with an excess of TAF7L express brown fat-specific genes and switch off muscle-specific genes. Further experiments suggested a role for TAF7L in connecting the core promoter of the gene with other DNA elements that regulate gene expression but lie some distance away from the promoter.

The work of Herrera et al. and Zhou et al. reinforces the idea that different TAFs provide the flexibility needed to control gene expression in a tissue-specific manner, and enable differentiating cells to change which genes they express rapidly. However many interesting questions remain: which signals lead to the destruction of core transcription factors? Are core promoter elements at tissue-specific genes designed to recognise variant TAFs? What determines whether variant TAFs are incorporated within TFIID, SAGA, or other complexes? It is known that TAF7 functions within TFIID to prevent transcription starting prematurely (Bhattacharya et al., 2014): does the TAF7L variant carry out a similar function at adipose tissue-specific genes?

Shortly after RNA polymerase II starts to transcribe a gene, it briefly pauses. Interestingly, a DNA sequence associated with this pausing, called the pause button, closely matches the sequences that bind to two subunits of TFIID (TAF6 and TAF9; Kadonaga, 2012). Consequently, TAF6 and TAF9 might be involved in pausing transcription**,** and if so, the variant TAF9B could play a similar role at motor neuron genes. Exploring the different patterns of gene expression in differentiated cells provides a powerful approach to help us to understand the logic of transcription.
